# What is the added value of patch testing with 30 fragrance allergens in addition to the European Baseline series?

**DOI:** 10.1111/cod.14065

**Published:** 2022-02-21

**Authors:** Rosalie C. Krijl, Norbertus A. Ipenburg, Sylvie M. Franken, Thomas Rustemeyer

**Affiliations:** ^1^ Department of Dermatology Amsterdam University Medical Centers Amsterdam The Netherlands

**Keywords:** 26 labeled fragrances, allergic contact dermatitis, contact allergy, cosmetics, fragrances, oxidized limonene, oxidized linalool, patch test, prehaptens

## Abstract

**Background:**

Patch testing with the fragrance allergy markers in the European baseline series (EBS) does not identify all patients with fragrance allergy. Hydroperoxides of linalool and limonene have been shown to be useful allergens in detecting fragrance sensitization.

**Objectives:**

To evaluate the added value of testing with 30 fragrance allergens in addition to the EBS.

**Methods:**

All patients with suspected fragrance allergy who underwent patch testing at the Amsterdam University Medical Centers between November 2019 and January 2021 to the EBS and fragrance series were included.

**Results:**

Of 323 patients tested, 162 (50.2%) were found to be fragrance sensitized. The most sensitizing single allergens were the hydroperoxides of linalool (1.0 and 0.5% pet.) and limonene (0.3 and 0.2% pet.). Testing with the hydroperoxides of linalool and limonene identified 62 fragrance‐sensitized patients (38.3%) who could not be detected by the common fragrance markers. Of all fragrance‐sensitized patients, 21 (13.0%) would have been missed when not testing with the fragrance series.

**Conclusions:**

Patch testing with the fragrance series in addition to the EBS is valuable. To reduce the risk of false‐negative reactions, it is advisable to test the hydroperoxides of linalool and limonene.

## Abstract 2

Highlights

- Of the 323 patients with suspected fragrance allergy, 162 (50.2%) were found to be fragrance sensitized.
- Testing with the hydroperoxides of linalool and limonene identified 62 fragrance‐sensitized patients (38.3%) who could not be detected by the European baseline series (EBS) fragrance markers. When not tested with the fragrance series, 21 (13.0%) of the sensitized patients would have been missed.
- Routinely performing patch testing with the fragrance series in addition to linalool and limonene hydroperoxide as part of the EBS is recommended.

## 1 INTRODUCTION

Fragranced substances are considered one of the most frequent causes of contact allergy.^1^, ^2^ In the general European population, sensitization to fragrances varies between 3.9% and 5.5% and has been increasing over the past decades.^2^, ^3^ This rising trend might be a consequence of the increased use of cosmetics and toiletries containing fragrances. Fragrances can also be found in cleansing agents, textiles, tobacco and in industrial settings.^4^ Due to these various applications, contact with fragrance‐related allergens remains hard to avoid.

Cosmetic products sold on the European market are required to be labeled if they contain a certain concentration of 26 fragrance substances known to be contact allergens in humans.^5^ Only 14 of these 26 substances are presented in the European baseline series (EBS) as part of the fragrance mixes I and II (FMI and FMII).^4^, ^6^ Previous studies showed that the EBS is not able to identify all patients with fragrance allergy.^7^, ^8^, ^9^, ^10^ The fragrance mixes may even fail to identify its own constituents.^7^, ^11^

As the exposure to environmental allergens is constantly changing over time, patch‐test series need to adapt as well in order to remain relevant.^12^ Common terpenes linalool and limonene are considered one of the most frequent fragrance ingredients.^13^ Moreover, dl‐limonene is also used as a solvent and industrial degreasing agent.^14^ Although they are uncommon fragrance allergens in their pure forms, oxidation transforms the prehaptens linalool and limonene into potent allergens.^15^, ^16^ These oxidized terpenes with stable concentrations of the main allergic hydroperoxides have been shown to be useful tools in detecting fragrance sensitization.^17^

Recently, various contact allergy groups have therefore advised the inclusion of oxidized linalool and dl‐limonene in the baseline series.^18^, ^19^ However, due to their irritant potential, inclusion is still under discussion concerning the EBS.^20^

Since 2019, the terpene hydroperoxides are routinely tested at the Amsterdam University Medical Centers (AUMC). In addition, oil of turpentine, a substance that is used as a raw material in the perfume industry, has been routinely tested due to increased sensitization rates.^21^ The aim of this study was to assess the added value of performing patch testing with the labeled fragrance substances, the hydroperoxides of linalool and limonene, and oil of turpentine in addition to the EBS. Other objectives were to report the sensitization rates of the tested allergens and to analyze co‐reactions between the fragrance allergens.

## 2 PATIENTS AND METHODS

### 2.1 Patients

In this retrospective cohort study, all patients with suspected fragrance allergy who had undergone patch testing with the EBS, the labeled fragrance substances, the hydroperoxides of linalool and limonene, and oil of turpentine (see Appendix 1 for all tested allergens) at the AUMC between November 2019 and January 2021 were analyzed. Patients who were <18 years of age and patients with angry‐back reactions were excluded. Approval was obtained from the Medical Ethics Review Committee of the Academic Medical Center (reference number W20_555 #20.618).

### 2.2 Patch testing

All patients were routinely tested with the EBS, the fragrance series of 26 (Trolab, Hermal, Reinbeck, Germany), oil of turpentine, and the hydroperoxides of linalool and limonene in each two dilutions (Chemotechnique Diagnostics, Vellinge, Sweden). The EBS contained *Myroxylon Pereirae*, hydroxyisohexyl 3‐cyclohexene carboxaldehyde (HICC), FMI and II. Sorbitan sesquioleate (SSO), used as an emulsifier in some allergen preparations,^22^ was also tested. Fragrance allergens containing SSO were FMI, *Myroxylon Pereirae*, *Evernia furfuracea*, *Evernia prunastri*, hydroxycitronellal, and isoeugenol (Table 1). The 26 individual fragrance substances that require labeling according to the EU Cosmetics Directive (including the pure forms of linalool and limonene) and oil of turpentine were considered as the fragrance series. Because HICC was included in the EBS as well as in the fragrance series, the fragrance series consist of 26 individual allergens that were tested in addition to the EBS when including oil of turpentine. The patch tests were performed with Van der Bend square chambers (Brielle, The Netherlands) applied on the upper part of the back for a period of 48 hours by using Fixomull stretch tape (Beiersdorf, Hamburg, Germany). Patch‐test preparations were supplied by Van der Bend (brands Trolab, Hermal, Reinbeck, Germany and Chemotechnique Diagnostics, Vellinge, Sweden). Patch‐test readings were executed 2, 3/4 and, if required, 6/7 days after application according to the recommendations of the European Society of Contact Dermatitis.^23^ The patch test reactions were scored as negative (−), questionable (?), irritant (IR), and allergic (+, ++, +++). All reactions + or higher were regarded as positive. Clinical relevance of positive patch‐test reactions was registered as certain, probable, possible, unlikely, or unknown. “Probable” and “certain” scores of relevance were considered as clinically relevant.

**TABLE 1:** Patch‐test reactions for EBS, the hydroperoxides of linalool and limonene, the 26 EU labeled fragrance substances, oil of turpentine, SSO, and colophonium

Allergen (all in pet.)	Positive n	Positive % (95%CI)	+	++	+++	?+	IR	Negative
Linalool hydroperoxide 1%^b^	78	24.1 (19.6‐29.2)	76 (23.5)	2 (0.6)	‐	117 (36.2)	1 (0.3)	127 (39.3)
Linalool hydroperoxide 0.5%^b^	56	17.3 (13.4‐21.9)	54 (16.7)	2 (0.6)	‐	54 (16.7)	‐	213 (65.9)
Fragrance mix I (FMI) 8%^a^ ^,^ ^f^	48	14.9 (11.2‐19.2)	38 (11.7)	10 (3.1)	‐	22 (6.8)	‐	253 (78.3)
Limonene hydroperoxide 0.2%^b^	45	13.9 (10.3‐18.2)	44 (13.6)	1 (0.3)	‐	42 (13.0)	‐	236 (73.1)
Myroxylon pereirae 25%^a^ ^,^ ^f^	36	11.1 (7.9‐15.1)	30 (9.3)	6 (1.9)	‐	21 (6.5)	1 (0.3)	265 (82.0)
Limonene hydroperoxide 0.3%^b^	33	10.2 (7.1‐14.0)	32 (9.9)	1 (0.3)	‐	36 (11.1)	2 (0.6)	252 (78.0)
Fragrance mix II (FMII) 14%^a^	26	8.0 (5.3‐11.6)	23 (7.1)	3 (0.9)	‐	19 (5.9)	‐	278 (86.1)
Citral 2%^c^ ^,^ ^e^	20	6.2 (3.8‐9.4)	19 (5.9)	1 (0.3)	‐	21 (6.5)	1 (0.3)	281 (87.0)
Colophonium 20%^g^	16	5.0 (2.9‐7.9)	16 (5.0)	‐	‐	4 (1.2)	1 (0.3)	302 (93.5)
*Evernia furfuracea* extract 1%^c^ ^,^ ^f^	15	4.6 (2.6‐7.5)	15 (4.6)	‐	‐	27 (8.4)	‐	281 (87.0)
*Evernia prunastri* extract 2%^c^ ^,^ ^d^ ^,^ ^f^	13	4.0 (2.2‐6.8)	12 (3.7)	1 (0.3)	‐	13 (4.0)	1 (0.3)	296 (91.6)
Isoeugenol 2%^c^ ^,^ ^d^ ^,^ ^f^	12	3.7 (1.9‐6.4)	10 (3.1)	2 (0.6)	‐	11 (3.4)	‐	300 (92.9)
Cinnamaldehyde 1%^c^ ^,^ ^d^	10	3.1 (1.5‐5.6)	9 (2.8)	‐	1 (0.3)	2 (0.6)	‐	311 (96.3)
HICC 5%^a^ ^,^ ^c^ ^,^ ^e^	8	2.5 (1.1‐7.9)	7 (2.2)	1 (0.3)	‐	10 (3.1)	‐	305 (94.4)
Cinnamyl alcohol 2%^c^ ^,^ ^d^	7	2.2 (0.9‐4.4)	7 (2.2)	‐	‐	2 (0.6)	1 (0.3)	313 (96.9)
Methyl 2‐octynoate 1%^c^	7	2.2 (0.9‐4.4)	6 (1.9)	1 (0.3)	‐	22 (6.8)	‐	294 (91.0)
Sorbitan sesquioleate (SSO)^h^	7	2.2 (0.9‐4.4)	6 (1.9)	1 (0.3)	‐	16 (5.0)	‐	300 (92.9)
Alpha‐isomethyl ionone 1%^c^	6	1.9 (0.7‐4.0)	6 (1.9)	‐	‐	4 (1.2)	‐	313 (96.9)
Benzyl salicylate 10%^c^	6	1.9 (0.7‐4.0)	6 (1.9)	‐	‐	18 (5.6)	‐	299 (92.6)
Citronellol 1%^c^ ^,^ ^e^	6	1.9 (0.7‐4.0)	6 (1.9)	‐	‐	11 (3.4)	‐	306 (94.7)
Farnesol 5%^c^ ^,^ ^e^	6	1.9 (0.7‐4.0)	6 (1.9)	‐	‐	6 (1.9)	‐	311 (96.3)
Geraniol 2%^c^ ^,^ ^d^	6	1.9 (0.7‐4.0)	6 (1.9)	‐	‐	13 (4.0)	‐	304 (94.1)
Benzyl cinnamate 5%^c^	5	1.5 (0.5‐3.6)	5 (1.5)	‐	‐	8 (2.5)	‐	310 (96.0)
Coumarin 5%^c^ ^,^ ^e^	5	1.5 (0.5‐3.6)	5 (1.5)	‐	‐	5 (1.5)	‐	313 (96.9)
dl‐limonene 2%^c^	5	1.5 (0.5‐3.6)	5 (1.5)	‐	‐	10 (3.1)	‐	308 (95.4)
Hydroxy‐citronellal 2%^c^ ^,^ ^d^ ^,^ ^f^	5	1.5 (0.5‐3.6)	5 (1.5)	‐	‐	4 (1.2)	‐	314 (97.2)
Eugenol 2%^c^ ^,^ ^d^	4	1.2 (0.3‐3.1)	3 (0.9)	1 (0.3)	‐	5 (1.5)	‐	314 (97.2)
Oil of turpentine 10%^c^	4	1.2 (0.3‐3.1)	3 (0.9)	1 (0.3)	‐	9 (2.8)	‐	310 (96.0)
Amyl cinnamal 2%^c^ ^,^ ^d^	3	0.9 (0.2‐2.7)	3 (0.9)	‐	‐	5 (1.5)	‐	315 (97.5)
Anisyl alcohol 1%^c^	3	0.9 (0.2‐2.7)	3 (0.9)	‐	‐	6 (1.9)	‐	314 (97.2)
Benzyl alcohol 1%^c^	3	0.9 (0.2‐2.7)	2 (0.6)	1 (0.3)	‐	9 (2.8)	1 (0.3)	310 (96.0)
Benzyl benzoate 10%^c^	3	0.9 (0.2‐2.7)	3 (0.9)	‐	‐	5 (1.5)	‐	315 (97.5)
Hexyl cinnamal 10%^c^ ^,^ ^e^	3	0.9 (0.2‐2.7)	3 (0.9)	‐	‐	15 (4.6)	‐	305 (94.4)
Linalool 10%^c^	3	0.9 (0.2‐2.7)	3 (0.9)	‐	‐	7 (2.2)	‐	313 (96.9)
Amylcinnamyl alcohol 1%^c^	2	0.6 (0.1‐2.2)	2 (0.6)	‐	‐	6 (1.9)	‐	315 (97.5)
Butylphenyl methylpropional 10%^c^	2	0.6 (0.1‐2.2)	1 (0.3)	1 (0.3)	‐	1 (0.3)	‐	320 (99.1)

*Note*: ?+, doubtful reaction; IR, irritant reaction; CI, confidence interval.

^a^ Part of baseline series.

^b^ Hydroperoxides of linalool and limonene.

^c^ Part of fragrance series.

^d^ Fragrance allergens constituting FM I.

^e^ Fragrance allergens constituting FM II.

^f^ Fragrance allergens containing SSO.

^g^ Colophonium is not part of the series but was added due to interest in positive reactions and co‐reactions to this substance.

^h^ SSO is not part of the series but is used as an emulsifier in FMI, *Myroxylon Pereirae*, *Evernia furfuracea*, *Evernia prunastri*, hydroxycitronellal, and isoeugenol.

### 2.3 Data collection

Prospectively collected data were extracted from the European Surveillance System on Contact Allergies (ESSCA) database of the AUMC and from electronic health records. The obtained data included demographics (sex, age), atopic history, symptoms (type, location), and patch‐test results (allergens applied, concentrations, reactions, and clinical relevance).

### 2.4 Statistical analysis

Statistical analyses were performed with SPSS, version 26.0.0.1 (IBM, SPSS, Chicago, IL).

Patient characteristics were presented as numbers and percentages for categorical variables and as medians with interquartile ranges (IQRs) for continuous variables. Demographic variables between groups were analyzed using χ^2^ tests or Mann‐Whitney test, as appropriate. The degree of association between categorical variables was measured by Spearman rank correlation. The correlation coefficient sizes (positive or negative) were interpreted as very *high* (0.9‐1.00), *high* (0.7‐0.9), *moderate* (0.5‐0.7), *low* (0.3‐0.5) and *negligible* (0.0‐0.3).^24^ Two‐sided *p*‐values of <0.05 were considered as statistically significant.

## 3 RESULTS

### 3.1 Patient characteristics

Between November 2019 and January 2021, a total of 344 patients suspected of having fragrance allergy were routinely patch tested with the EBS, hydroperoxides of linalool and limonene, and the fragrance series. Of these, 21 patients (6.1%) had not yet reached the age of 18 on the first day of testing and were therefore excluded. In total, 323 patients could be included in this study. The majority of these patients was female (75.9%) with a median age of 41 years (interquartile range [IQR]: 30‐54). Fragrance‐sensitized patients were significantly more likely than nonsensitized patients to have face dermatitis (*p* = 0.02) (Appendix 3).

### 3.2 Patch testing

A total of 502 positive reactions were seen in 162 patients (50.2%) (emulsifier SSO not included). Among the individual allergens, sensitization was most frequent for linalool hydroperoxide 1.0% and 0.5% pet. (24.1% and 17.2%, respectively) and limonene hydroperoxide 0.3% and 0.2% pet. (10.2% and 13.9%, respectively) (Table 1). All allergens of the fragrance series elicited positive patch‐test reactions. Among these, the most sensitizing allergens were citral (6.2%), *Evernia furfuracea* **(**4.6%), and *Evernia prunastri (*4.0%). Positive patch tests to butylphenyl methylpropional and amylcinnamyl alcohol (both 0.6%) were least common. Doubtful reactions were observed for all allergens but most frequently for linalool and limonene hydroperoxide (Table 1). Irritant reactions were recorded for *Myroxylon pereirae*, linalool hydroperoxide 1.0% pet., limonene hydroperoxide 0.3% pet., cinnamyl alcohol, *Evernia prunastri*, citral, and benzyl alcohol. Co‐reactivity to SSO among the fragrance allergens containing SSO were 3 of the 48 (6.3%) positive patch tests to FMI and 1 of the 13 (7.7%) and 1 of the 15 (6.7%) positive patch tests to *Evernia prunastri* and *Evernia furfuracea*, respectively. The fragrance allergens *Myroxylon pereirae*, hydroxycitronellal, and isoeugenol did not yield concomitant positive patch‐test reactions to SSO.

### 3.3 Clinical relevance

The clinical relevance could not be assessed for 41.8% of all positive patch‐test reactions. Of the assessed reactions, 58.9% were considered clinically relevant. Sensitization to HICC (80.0%), FMII (69.5%), FMI (62.5%), *Myroxylon pereirae* (56.5%), and the hydroperoxides of linalool 1% pet. (55.1%) and 0.5% pet. (58.3%) and limonene 0.3% pet. (55.6%) and 0.2% pet. (50.0%) were most frequently considered as clinically relevant.

### 3.4 Test series

**FIGURE 1:**
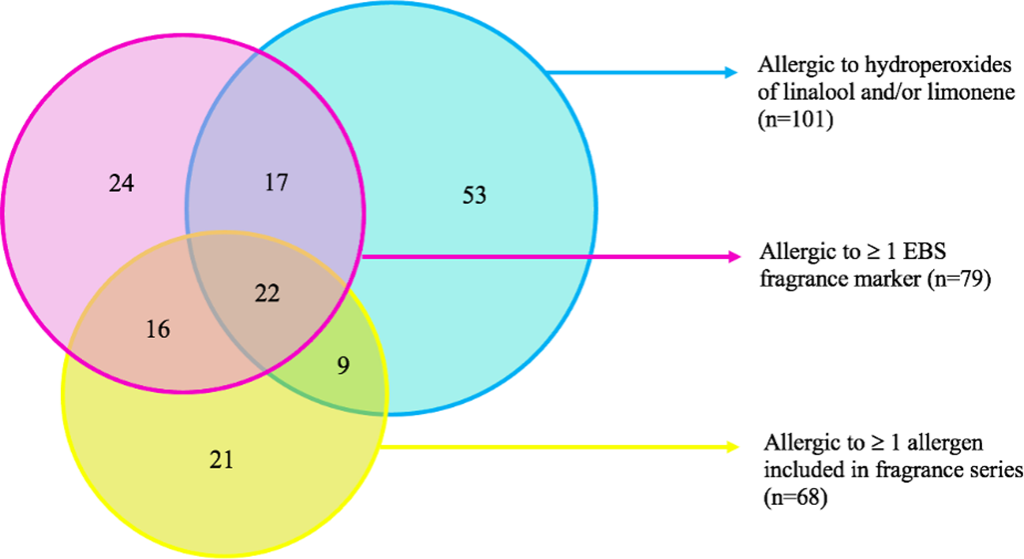
Venn diagram of the sensitized patients (n = 162) distributed over the patch‐test series that identified their allergies: Fragrance markers included in the European baseline series (EBS; pink), the hydroperoxides of linalool and limonene (cyan), and the fragrance series (yellow). The overlapping parts represent concomitant co‐reactions of the patch‐test series

Of all 502 positive patch‐test reactions, 330 (65.7%) were reactions to allergens in the EBS. In total, 172 reactions (34.3%) were identified by testing the fragrance series. Linalool hydroperoxide (1% and 0.5% pet.) and limonene hydroperoxide (0.3% and 0.2% pet.), accounted for 212 positive patch tests in 101 patients (31.3%). Of the 162 fragrance‐sensitized patients, 94 (58.0%) had their allergies fully defined by the EBS alone and 21 (13.0%) fully by the fragrance series alone (Figure 1). In 53 patients (32.7%), the oxidized forms of linalool and limonene were the only allergens that yielded positive patch‐test reactions. Forty‐seven patients (29.0%) tested positive to the EBS as well as the fragrance series but in only 13 of these patients (27.7%) could the allergies be fully explained by the EBS.

### 3.5 FMI, FMII, and their constituents

Of the 48 patients who had positive patch‐test reactions to FMI, 25 patients (52.1%) were also sensitized to at least one of the single constituents of the mix. Concerning FMII, this was the case for 10 of 26 patients (38.5%) (Table 2). Twenty‐five (65.8%) concomitant positive patch tests to FMI were recorded among the 38 patients who were sensitized to at least one of the single constituents of FMI. The concomitant reactivity to FMII in patients sensitized to the constituents of the mix was 32.3% (10 of the 31 patients) (Table 3). Among the seven patients sensitized to SSO, three concomitant reactions were reported to both FMI and FMII. One of the FMI‐sensitized patients and two of the FMII‐sensitized patients had positive reactions to the constituents of the mix.

**TABLE 2:** Positive patch‐test results to the EBS and the hydroperoxides of linalool and limonene with concomitant positive reactions to the fragrance series

Allergen	Positive patch tests	Co‐reactions with fragrance series (%)	Co‐reactions with FM1 constituents (%)	Co‐reactions with FMII constituents (%)
Fragrance mix I (FMI)	48	30 (62.5)	25 (52.1)	15 (31.3)
Fragrance mix II (FMII)	26	14 (53.8)	8 (30.8)	10 (38.5)
Myroxylon pereirae	36	18 (50.0)	12 (33.3)	12 (33.3)
HICC	8	8 (100)	6 (75.0)	7 (87.5)
Linalool hydroperoxide 1%	78	25 (32.1)	14 (17.9)	12 (15.4)
Linalool hydroperoxide 0.5%	56	20 (35.7)	12 (21.4)	11 (19.6)
Limonene hydroperoxide 0.3%	33	13 (39.4)	9 (27.3)	9 (27.3)
Limonene hydroperoxide 0.2%	45	16 (35.6)	10 (22.2)	10 (22.2)
Sorbitan sesquioleate (SSO)^a^	7	3 (42.6)	1 (14.3)	3 (42.6)

^a^ SSO is not part of the baseline series but is used as an emulsifier in FMI, *Myroxylon Pereirae*, *Evernia furfuracea*, *Evernia prunastri*, hydroxycitronellal, and isoeugenol.

**TABLE 3:** Positive patch‐test results to the fragrance series with co‐reactions to the EBS (FMI, FMII, *M.pereirae*, HICC), fragrance mix I (FMI), and fragrance mix II (FMII)

Allergen	Positive patch test reactions	Co‐reactions with EBS (%)	Co‐reactions to FMI (%)	Co‐reactions to FMII (%)
Amyl cinnamal^a^	3	2 (66.7)	2 (66.7)	0 (0)
Cinnamyl alcohol^a^	7	6 (85.7)	6 (85.7)	2 (28.6)
Eugenol^a^	4	4 (100)	4 (100)	2 (50.0)
Hydroxycitronellal^a^	5	4 (80.0)	3 (60.0)	2 (40.0)
Isoeugenol^a^	12	11 (91.7)	9 (75.0)	4 (33.3)
Cinnamaldehyde^a^	10	9 (90.0)	8 (80.0)	3 (30.0)
Geraniol^a^	6	6 (100)	5 (83.3)	6 (100)
*Evernia prunastri* extract^a^	13	10 (76.9)	8 (61.5)	1 (7.7)
*At least one FMI constituent*	38	30 (78.9)	25 (65.8)	8 (21.1)
Citral^b^	20	16 (84.2)	9 (47.4)	7 (36.8)
HICC^b^	8	8 (100)	7 (87.5)	5 (62.5)
Farnesol^b^	6	6 (100)	5 (83.3)	4 (66.7)
Coumarin^b^	5	3 (60.0)	1 (20.0)	1 (20.0)
Citronellol^b^	6	5 (83.3)	4 (66.7)	2 (33.3)
Hexyl cinnamaldehyde^b^	3	2 (66.7)	2 (66.7)	0 (0)
*At least one FMII constituent*	31	24 (77.4)	15 (48.4)	10 (32.3)
Oil of turpentine	4	3 (75.0)	3 (75.0)	1 (25.0)
Benzyl alcohol	3	2 (66.7)	2 (66.7)	0 (0)
Benzyl salicylate	6	5 (83.3)	2 (33.3)	0 (0)
Benzyl cinnamate	5	3 (60.0)	4 (80.0)	1 (20.0)
Benzyl benzoate	3	2 (66.7)	1 (33.3)	1 (33.3)
Dl‐limonene	5	4 (80.0)	3 (60.0)	1 (20.0)
*Evernia furfuracea* extract	15	10 (66.7)	7 (46.7)	2 (13.3)
Amylcinnamyl alcohol	2	2 (100)	1 (50.0)	0 (0)
Anise alcohol	3	3 (100)	1 (33.3)	1 (33.3)
Butylphenyl methylpropional	2	2 (100)	0 (0)	1 (50.0)
Alpha‐isomethyl ionone	6	3 (50.0)	2 (33.3)	1 (16.7)
Linalool	3	3 (100)	2 (66.7)	1 (33.3)
Methyl 2‐octynoate	7	6 (85.7)	4 (57.1)	0 (0)
Total	172	140 (81.4)	105 (61.0)	49 (28.5)
Sorbitan sesquioleate (SSO)^c^	7	5 (71.4)	3 (42.9)	3 (42.9)

^a^ Fragrance allergens constituting FMI.

^b^ Fragrance allergens constituting FMII.

^c^ SSO is not part of the baseline series but is used as an emulsifier in FMI, *Myroxylon pereirae*, *Evernia furfuracea*, *Evernia prunastri*, hydroxycitronellal, and isoeugenol.

### 3.6 Linalool and limonene

Of the patients sensitized to linalool hydroperoxide 1.0% pet., 60.3% had a co‐reaction to its less concentrated form (0.5% pet.). All three patients positive to unoxidized linalool also patch tested positive to oxidized linalool. Only two of the five patients who tested positive to unoxidized limonene had concomitant positive patch‐test reactions to its oxidized form. Of the patients sensitized to limonene hydroperoxide (0.3% pet.), 93.9% had a concomitant reaction to the less concentrated form (0.2% pet.). Concomitant reactions to the fragrance series in patients sensitized to oxidized linalool or oxidized limonene varied between 32.1% and 39.4% (Table 2). The role of the oxidized terpenes as fragrance allergy markers was measured in a correlation analysis. No relevant significant associations with other individual allergens or markers were found (Appendix 2).

### 3.7 Oil of turpentine

Oil of turpentine yielded a sensitization rate of 1.2%, as four patients reacted positive. Seventy‐five percent of the patients with positive patch‐test reactions to oil of turpentine had concomitant positive patch‐test reactions to FMI, *Myroxylon pereirae*, and linalool hydroperoxide 0.5% pet. Concomitant reactions to both dilutions of linalool and limonene hydroperoxide were present in two (50.0%) of the four sensitized patients. Allergens that yielded one concomitant reaction were FMII, HICC, citral, hydroxycitronellal, eugenol, *Evernia prunastri*, isoeugenol, cinnamaldehyde, and benzyl cinnamate.

## 4 DISCUSSION

In this retrospective cohort study, the added value of testing with the hydroperoxides of linalool and limonene, the EU‐labeled fragrance allergens, and oil of turpentine, in addition to the EBS, was evaluated. If only the EBS was tested, 34.0% of the sensitized patients would not have had their allergies fully defined and 13.0% would have remained undetected as fragrance allergic. Without patch testing with the hydroperoxides of linalool and limonene, 38.3% of all fragrance‐sensitized patients would be missed. Therefore, it is valuable to perform patch testing with the fragrance series in addition to the EBS. Patch testing with the hydroperoxides of linalool and limonene in the EBS will reduce the risk of false‐negative reactions, vs testing with nonoxidized linalool and limonene.

### 4.1 Fragrance sensitization

Fragrance sensitization was present in about half of our patch‐tested patients (50.2%) and was significantly associated with facial dermatitis. Lower rates of fragrance allergy (7.6%‐ 26.9%) were reported in previous studies.^8^, ^9^, ^10^, ^25^ Referral bias and selection bias might be the cause of this difference. In addition, the individual constituents of FMI (except for cinnamal) in this study were patch tested at double the concentrations used than in two previous studies.^10^, ^25^ By testing at higher concentrations, an improved diagnostic ability is expected.^6^, ^11^, ^26^, ^27^ In one previous study, the hydroperoxides of linalool (1% pet.) and limonene (0.3% pet.) were also tested.^25^ The higher sensitization rate in the current study (50.2% vs 15.7%) can be explained partly by patch testing linalool and limonene hydroperoxide in the two recommended dilutions instead of one.^20^ Besides that, *Myroxylon pereirae* and methyl‐2‐octynate were not included as fragrance allergens in the Danish study.

### 4.2 Linalool and limonene

As expected, the hydroperoxides of linalool 1.0% and 0.3% pet. and limonene 0.3% and 0.2% pet. were among the most sensitizing (24.1%, 17.2%, 10.2%, and 13.9% respectively).^15^, ^18^, ^25^, ^28^, ^29^ Comparable sensitization rates were reported for linalool hydroperoxide 1% pet. (19.8%) and limonene hydroperoxide 0.3% pet. (7.6%) in a retrospective study performed in the United States.^28^ Linalool hydroperoxide 1% pet. was able to detect most of the linalool allergies. In addition, it yielded most of the questionable and irritant reactions. In contrast, most of the positive and irritative reactions were identified by the less concentrated form of limonene hydroperoxide (0.2% pet.). In dose‐finding studies, the highest concentrations of linalool and limonene hydroperoxides were able to detect most of the sensitized patients.^17^, ^18^ Therefore, we cannot find an explanation for limonene hydroperoxide 0.2% pet. detecting most of the limonene allergies. Although irritative reactions to these terpenes are relatively common, it is worth including them in the EBS given the number of fragrance‐sensitized patients detected. In addition, the relevance scores of these substances were among the highest. The clinical relevance of contact allergy to limonene hydroperoxide in patients with positive patch‐test reactions was also emphasized in a recent validation repeated open application test (ROAT) study.^30^ To aid in patch‐test interpretation concerning questionable and irritative reactions, the European Society of Contact Dermatitis has recently recommended testing in both dilutions.^20^

### 4.3 FMI and FMII

Rates of concomitant sensitivity among patients positive to the fragrance series were 61.0% to FMI and 28.5% to FMII. The higher concomitant sensitivity to FMI than to FMII is in line with previous reports.^8^, ^9^, ^10^ Of the patients sensitized to FMI and FMII, concomitant reactions to the constituents were present in 52.1% and 38.5%, respectively. In contrast, more concomitant reactions were reported between FMII and its constituents in a recent Danish study.^25^ As mentioned before, the constituents of FMI (except for cinnamal) were prepared at half of the concentration used in the current study. A negative breakdown test to FMI does not rule out contact allergy to its constituents. When adequate concentrations are not patch tested, false‐negative reactions to FMI constituents may be expected.^11^

### 4.4 Individual fragrance allergens

Among the EU‐labeled allergens, citral (6.2%), *Evernia furfuracea* (4.6%), and *Evernia prunastri* (4.0%) were the most sensitizing. This is in line with previous reports, especially for the last two allergens.^8^, ^9^, ^10^, ^25^, ^29^, ^31^ The high sensitization rate of *Evernia prunastri* is attributable mostly to its allergic compounds chloroatranol and atranol.^32^ Due to their hyperallergenic potential, cosmetics containing these compounds are no longer allowed to be sold in the EU from August 2021.^33^, ^34^ The patients sensitized to *Evernia furfuracea* can be divided into two subgroups: those with sensitization to common constituents as also present in *Evernia prunastri*, and those sensitized to oxidized resin acids as present in the contaminating tree bark which is used in the extraction process. This latter subgroup was identified by positive reactions to colophonium.^35^ Five of the 15 patients (33.3%) sensitized to *Evernia furfuracea* had positive co‐reactions to colophonium. Two of these double‐sensitized patients (40%) reacted negative to *Evernia prunastri*. The high co‐sensitization rate to colophonium among patients sensizited to *Evernia* furfuracea in combination with the fact that not all patients were sensitized to *Evernia prunastri* supports the heterogeneity in this sensitized group.

Citral is a mixture of the two aldehydes geranial and neral. These compounds have also been found in the oxidation process of geraniol. Just like linalool and limonene, geraniol has the potential to form allergenic compounds by autooxidation on air exposure.^36^ Because co‐reactions between citral or its components and oxidized geraniol are recently specified in literature, sensitization to citral can be explained partly by this co‐reactivity.^37^ In a recent Swedish study, oxidized geraniol yielded a significantly higher sensitization rate than its pure form.^38^ Patch testing with oxidized geraniol may therefore be of value. Due to oxidized geraniol currently being unavailable for patch testing, this cannot be confirmed by the current study. Oil of turpentine was tested as well, owing to the interest in this substance as a fragrance allergen, and yielded a sensitization rate of 1.2%. Seventy‐five percent of the patients with positive patch‐test reactions to oil of turpentine had concomitant positive patch‐test reactions to fragrance markers: FMI, *Myroxylon pereirae*, and a recommended addition to the EBS: linalool hydroperoxide 0.5% pet. Only one of the four sensitized cases in the current study remained undetected by the fragrance markers; therefore, the added value of patch testing this substance consecutively is questionable.

### 4.5 Limitations

Selection bias might have been introduced by including patients suspected of having fragrance allergies. Referral bias and missing clinical relevance data are limitations of this study. In addition, the sources of exposure were not investigated.

## 5 CONCLUSION

In conclusion, testing with the hydroperoxides of linalool and limonene in each two dilutions has been useful to identify fragrance‐sensitized patients who could not be detected by the common markers. Testing with the fragrance series of 26 detects even more fragrance‐allergic cases and can help in advising what allergens to avoid. We recommend routinely performing patch testing with the fragrance series in addition to linalool and limonene hydroperoxide as part of the EBS in all patients with suspected fragrance allergy. Future studies are needed to evaluate the possible added value of testing the oxidized form of geraniol.

## CONFLICTS OF INTEREST

None declared

## AUTHOR CONTRIBUTIONS

**Rosalie C. Krijl:** Conceptualization (lead); formal analysis (lead); investigation (lead); methodology (lead); visualization (lead); writing – original draft (lead); writing – review & editing (lead). **Norbertus A. Ipenburg:** Conceptualization (lead); data curation (equal); investigation (supporting); methodology (supporting); project administration (lead); resources (lead); writing – review and editing (lead). **Sylvie M. Franken:** Conceptualization (supporting); data curation (equal); writing – review and editing (supporting). **Thomas Rustemeyer:** Conceptualization (supporting); Data curation (equal); Methodology (supporting); Supervision (Lead); Visualization (supporting); Writing – review & editing (supporting).

## Supporting information


**Appendix S1.** **The fragrance allergens per test series included for this study.**
**Appendix S2.** Correlation analysis between the oxidized forms of linalool and limonene and the fragrance allergens from the European baseline series and fragrance series. Spearman rank correlation (R) along with its *p*‐values are tabulated.
**Appendix S3.** Demographic characteristics according to the MOAHLFA index for all patients tested

## Data Availability

Research data are not shared
