# Supplementary material for: What is the added value of patch testing with 30 fragrance allergens in addition to the European Baseline series?
Source: Contact Dermatitis. 2022 Feb 21;86(5):390–7. doi: 10.1111/cod.14065 (PMC9302649; doi:10.1111/cod.14065)
Supplement: Supplementary file 1 — Appendix S1. The fragrance allergens per test series included for this study. Appendix S2. Correlation analysis between the oxidized forms of linalool and limonene and the fragrance allergens from the European baseline series and fragrance series. Spearman rank correlation (R) along with its p‐values are tabulated. Appendix S3. Demographic characteristics according to the MOAHLFA index for all patients tested [file COD-86-390-s001.docx]

**Appendix**

**Appendix 1: The fragrance allergens per test series included for this study.**

1. **European Baseline Series (EBS) fragrance allergens. All allergens were dissolved in petrolatum.**

| number | *allergen* | *concentration* |
| --- | --- | --- |
| 1. | Myroxylon pereirae | 25% |
| 2. | Fragrance mix I | 8% |
| 3. | Fragrance mix II | 14% |
| 4. | Hydroxyisohexyl 3-cyclohexene carboxaldehyde (HICC)† | 5% |

1. **The 30 fragrance allergens tested in addition to the EBS. All allergens were dissolved in petrolatum.**

| number | *allergen* | *concentration* |
| --- | --- | --- |
| 1. | Linalool hydroperoxide | 1% |
| 2. | Linalool hydroperoxide | 0.5% |
| 3. | Limonene hydroperoxide | 0.3% |
| 4. | Limonene hydroperoxide | 0.2% |
| 5. | Amyl cinnamal†‡ | 2% |
| 6. | Cinnamyl alcohol†‡ | 2% |
| 7. | Eugenol†‡ | 2% |
| 8. | Hydroxycitronellal†‡ | 2% |
| 9. | Isoeugenol†‡ | 2% |
| 10. | Cinnamaldehyde†‡ | 1% |
| 11. | Geraniol†‡ | 2% |
| 12. | Evernia prunastri extract†‡ | 2% |
| 13. | Citral†§ | 2% |
| 14. | Farnesol†§ | 5% |
| 15. | Citronellol†§ | 1% |
| 16. | Hexyl cinnamal†§ | 10% |
| 17. | Benzyl alcohol† | 1% |
| 18. | Benzyl salicylate† | 10% |
| 19. | Benzyl cinnamate† | 5% |
| 20. | Benzyl benzoate† | 10% |
| 21. | dl-Limonene† | 2% |
| 22. | Evernia furfuracea extract† | 1% |
| 23. | Amylcinnamyl alcohol† | 1% |
| 24. | Anise alcohol† | 1% |
| 25. | Butylphenyl Methylpropional† | 10% |
| 26. | Alpha-isomethyl ionone† | 1% |
| 27. | Coumarin†§ | 5% |
| 28. | Linalool† | 10% |
| 29. | Methyl 2-octynoate† | 1% |
| 30. | Oil of Turpentine† | 10% |

†Tested as part of the fragrance series, ‡Fragrance allergens constituting FM I, §Fragrance allergens constituting FM II.

**Appendix 2.** Correlation analysis between the oxidized forms of linalool and limonene and the fragrance allergens from the EBS and fragrance series. Spearman rank correlation (R) along with its p-values are tabulated.

| Allergen marker | Linalool hydroperoxide 1%  Correlation coefficient (p-value) | Linalool hydroperoxide 0.5%  Correlation coefficient (p-value) | Limonene hydroperoxide 0.3%  Correlation coefficient (p-value) | Limonene hydroperoxide 0.2%  Correlation coefficient (p-value) |
| --- | --- | --- | --- | --- |
| Fragrance mix I (FMI) | 0.090 (0.108) | 0.177 (0.001)** | 0.175 (0.002)** | 0.159 (0.004)** |
| Fragrance mix II (FMII) | 0.126 (0.024)* | 0.105 (0.059) | 0.163 (0.003)** | 0.177 (0.001)** |
| Myroxylon pereirae | 0.260 (0.00)** | 0.306 (0.00)** | 0.303 (0.00)** | 0.312 (0.00)** |
| Hydroxyisohexyl 3-cyclohexene carboxaldehyde (HICC) | 0.143 (0.010)* | 0.137 (0.013)* | 0.209 (0.00)** | 0.166 (0.003)** |
| Amyl cinnamal† | -0.055 (0.328) | -0.044 (0.427) | -0.033 (0.559) | -0.039 (0.485) |
| Cinnamyl alcohol† | 0.015 (0.783) | -0.012 (0.830) | 0.020 (0.720) | 0.002 (0.978) |
| Eugenol† | 0.133 (0.017)* | 0.171 (0.002)** | 0.055 (0.327) | 0.117 (0.036) |
| Hydroxycitronellal† | 0.046 (0.405) | 0.075 (0.178) | 0.206 (0.00)** | 0.167 (0.003)** |
| Isoeugenol† | 0.119 (0.033)* | 0.126 (0.023)* | 0.096 (0.085) | 0.110 (0.048)* |
| Cinnamaldehyde† | -0.059 (0.290) | 0.013 (0.822) | 0.117 (0.036)* | 0.083 (0.137) |
| Geraniol† | 0.030 (0.597) | 0.058 (0.298) | 0.181 (0.001)** | 0.143 (0.010)** |
| Evernia prunastri extract† | 0.032 (0.571) | 0.114 (0.04)* | 0.139 (0.012)* | 0.100 (0.074) |
| Citral‡ | 0.065 (0.243) | 0.120 (0.031)* | 0.125 (0.024)* | 0.119 (0.032) |
| Farnesol‡ | 0.030 (0.597) | -0.002) (0.965) | 0.029 (0.600) | 0.077 (0.167) |
| Coumarin‡ | 0.046 (0.405) | 0.075 (0.178) | 0.206 (0.00)** | 0.167 (0.003)** |
| Citronellol‡ | 0.030 (0.597) | 0.058 (0.298) | 0.105 (0.059) | 0.077 (0.167) |
| Hexyl cinnamal‡ | 0.021 (0.710) | 0.041 (0.464) | 0.074 (0.185) | 0.054 (0.331) |
| Oil of Turpentine | 0.068 (0.225) | 0.171 (0.002)** | 0.147 (0.008)** | 0.117 (0.036)* |
| Benzyl alcohol | 0.021 (0.710) | 0.041 (0.464) | 0.074 (0.185) | 0.054 (0.331) |
| Benzyl salicylate | 0.083 (0.136) | -0.002 (0.965) | 0.029 (0.600) | 0.077 (0.167) |
| Benzyl cinnamate | 0.046 (0.405) | 0.075 (0.178) | 0.041 (0.468) | 0.022 (0.694) |
| Benzyl benzoate | 0.021 (0.710) | 0.041 (0.464) | 0.180 (0.001)** | 0.147 (0.008)** |
| dl-Limonene | -0.012 (0.828) | 0.009 (0.875) | 0.123 (0.027)* | 0.094 (0.090) |
| Evernia furfuracea extract | 0.047 (0.396) | 0.054 (0.330) | 0.217 (0.00)** | 0.166 (0.003)** |
| Amylcinnamyl alcohol | 0.140 (0.012)* | 0.068 (0.222) | 0.104 (0.063) | 0.082 (0.140) |
| Anise alcohol | 0.096 (0.084) | 0.041 (0.464) | 0.180 (0.001)** | 0.147 (0.008)** |
| Butylphenyl Methylpropional | -0.045 (0.425) | -0.036 (0.517) | 0.104 (0.063) | 0.196 (0.000)** |
| Alpha-isomethyl ionone | 0.030 (0.597) | -0.002 (0.965) | 0.105 (0.059) | 0.077 (0.167) |
| Linalool | 0.172 (0.002)** | 0.126 (0.023)* | 0.180 (0.001)** | 0.147 (0.008)** |
| Methyl 2-octynoate | 0.065 (0.244) | 0.1 (0.072) | 0.020 (0.720 | 0.063 (0.259) |

†Fragrance allergens constituting FM I, ‡Fragrance allergens constituting FM II.

*correlation is significant at the 0.05 level (2-tailed)

** correlation is significant at the 0.01 level (2-tailed)

**Appendix 3.** Demographic characteristics according to the MOAHLFA index for all patients tested

|  | All patients  N=323 (%) | Fragrance negative  N= 161 (%) | Fragrance positive  N= 162 (%) | Fragrance positive versus fragrance negative, p value † |
| --- | --- | --- | --- | --- |
| Age (years), median, (IQR) | 41 (24) | 39 (23) | 43 (23) | 0.97 |
| Male, n (%) | 78 (24.1) | 38 (23.6) | 40 (24.7) | 0.82 |
| Occupational, n (%) | 45 (13.9) | 17 (10.6) | 28 (17.3) | 0.08 |
| Atopy, n (%) | 206 (63.8) | 106 (65.8) | 100 (61.7) | 0.44 |
| Hand dermatitis, n (%) | 83 (25.7) | 35 (21.7) | 48 (29.6) | 0.11 |
| Leg dermatitis, n (%) | 68 (21.1) | 30 (18.6) | 38 (23.5) | 0.29 |
| Face dermatitis, n (%) | 142 (44.0) | 60 (37.3) | 82 (50.6) | 0.02* |
| Age ≥ 40 years, n (%) | 171 (52.9) | 80 (49.7) | 91 (56.2) | 0.24 |

IQR, interquartile range

†Chi *^2^*test was used for categorical variables and Mann-Whitney for U-test for continuous variables

* correlation is significant at the 0.05 level (2-tailed)
